# Longitudinal cervicovaginal microbiome and virome alterations during ART and discordant shedding in women living with HIV

**DOI:** 10.21203/rs.3.rs-4078561/v1

**Published:** 2024-04-15

**Authors:** Emily A. Kaelin, Caroline Mitchell, Jamie Soria, Alberto La Rosa, Eduardo Ticona, Robert W. Coombs, Lisa M. Frenkel, Marta E. Bull, Efrem S. Lim

**Affiliations:** Arizona State University; University of Washington; Universidad de San Marcos; Asociaciòn Civil Impacta Salud y Educatión; Universidad de San Marcos; University of Washington; University of Washington; Seattle Children’s Research Institute; Arizona State University

## Abstract

Despite successful suppression of plasma HIV replication by antiretroviral therapy (ART), some women living with HIV (WLHIV) can still experience genital HIV shedding (discordant shedding). Female genital tract (FGT) microbiome and virome dynamics during long-term ART in WLHIV are poorly understood but might contribute to discordant HIV shedding, as the microbiome and virome are known to influence FGT health. To understand FGT microbial communities over time during ART usage and discordant shedding, we characterized the microbiome and virome in 125 cervicovaginal specimens collected over two years in 31 WLHIV in Lima, Peru. Intrapersonal bacterial microbiome variation was higher in HIV shedders compared to non-shedders. Cervicovaginal virome composition changed over time, particularly in non-shedders. Specifically, anellovirus relative abundance was inversely associated with ART duration and CD4 counts. Our results suggest that discordant HIV shedding is associated with FGT microbiome instability, and immune recovery during ART influences FGT virome composition.

## Introduction

With the development of effective antiretroviral therapies (ART), life expectancy for people living with HIV has increased significantly and HIV infection can now be treated as a chronic disease^1,2^. Of the estimated 20 million women aged 15 and older living with HIV worldwide in 2022, an estimated 82% were taking ART^3^. While ART is effective in suppressing HIV replication, some women living with HIV (WLHIV) with undetectable plasma viral loads can still experience genital HIV shedding, described as discordant shedding^4-10^. The microbiome has been implicated in female genital tract (FGT) health and disease^11^. However, the relationship between discordant shedding and the FGT microbiome and virome during long-term ART in WLHIV remains poorly understood.

The vaginal microbiomes of reproductive-aged women typically consist mainly of *Lactobacillus* or diverse anaerobes including *Gardnerella, Prevotella, Sneathia, Fannyhessea, Megasphaera, Dialister, Mobiluncus, Peptonophilus, Shuttleworthia, Parvimonas* and other genera^11-15^. Lactobacilli contribute to vaginal health by producing lactic acid, which lowers vaginal pH and creates a less favorable environment for pathogenic bacteria, and by competing with pathogens for space^11^. Cervicovaginal microbiome alterations have been associated with inflammation^16^, preterm birth^17^, changes to the mucosal epithelial barrier^18^, cervical cancer^19,20^, and sexually transmitted infections^14,20-22^. Although higher *Lactobacillus* relative abundance has been associated with vaginal health, many asymptomatic women have diverse vaginal microbiomes dominated by bacteria other than lactobacilli^12-15^. Longitudinal studies have demonstrated that the vaginal microbiome is stable over time in some women, while in others vaginal microbiome composition fluctuates more frequently^13,16^.

The FGT microbiome is particularly important in HIV infection. Specific microbiome community states and bacterial species have been associated with increased HIV prevalence^22^ and acquisition^14^. In WLHIV, cervicovaginal microbiota have been associated with genital HIV shedding^5,22,23^. While pre-exposure prophylaxis (PrEP) has not been shown to significantly influence the vaginal microbiome in women without HIV^24-26^, multiple lines of evidence suggest that vaginal bacteria influence metabolism and concentrations of antiretroviral drugs in the FGT^24,27-32^. Whether long-term ART influences the vaginal microbiome in WLHIV is poorly understood. One study found no microbiome changes after six months of ART^33^, another reported higher *Lactobacillus* relative abundance in women with viral suppression after a year of ART^34^, and a third reported an association between bacterial vaginosis and ART with protease inhibitors compared to regimens without protease inhibitors^35^. Further study is needed to clarify the relationship between ART and the FGT microbiome over years or decades. The relationship between the microbiome and discordant HIV shedding is also unclear. Discordant HIV shedding is incompletely understood, but has been associated with genital inflammation^36^, coinfections^36,37^, trauma^38^ and ulcers^10^, and has also been attributed to expression of integrated provirus from proliferating cells^4^. The importance of the microbiome in FGT health led us to consider the microbiome as a potential factor promoting discordant HIV shedding.

Compared to the bacterial microbiome, less is known about the role of the virome in FGT health. Viruses found in the FGT include papillomaviruses, anelloviruses, herpesviruses and bacteriophages^39-47^. Vaginal virome community structure has been linked with the bacterial microbiome^40-42^. Vaginal virome alterations have been associated with inflammation^41^, cervical lesions^43,44^, and preterm birth^45^. Human papillomaviruses are the major cause of cervical cancer, the fourth most-diagnosed cancer in women worldwide in 2020^48^. Anelloviruses, a recently discovered, prevalent viral family^49^, have been associated with FGT inflammation in women without HIV^41^ and CD4 T cell levels in WLHIV^44^. Little is known about the relationship between the FGT virome and long-term ART or discordant HIV shedding.

To investigate the relationship between the cervicovaginal microbiome and virome and discordant shedding over time in WLHIV taking ART, we analyzed the microbiome and virome in 125 cervicovaginal specimens collected over a two-year period from 31 WLHIV in Lima, Peru, nine of whom experienced discordant HIV shedding. Intrapersonal bacterial microbiome stability differed significantly between HIV shedders and non-shedders. Significant virome changes were associated with ART duration, specifically decreases in anellovirus abundance, especially among non-shedders. Our results suggest that cervicovaginal microbiome instability is associated with discordant HIV shedding, while immune recovery during ART may influence the cervicovaginal virome.

## RESULTS

### Shedders’ bacterial microbiomes are less stable over time than non-shedders’ microbiomes.

**Figure 1:**
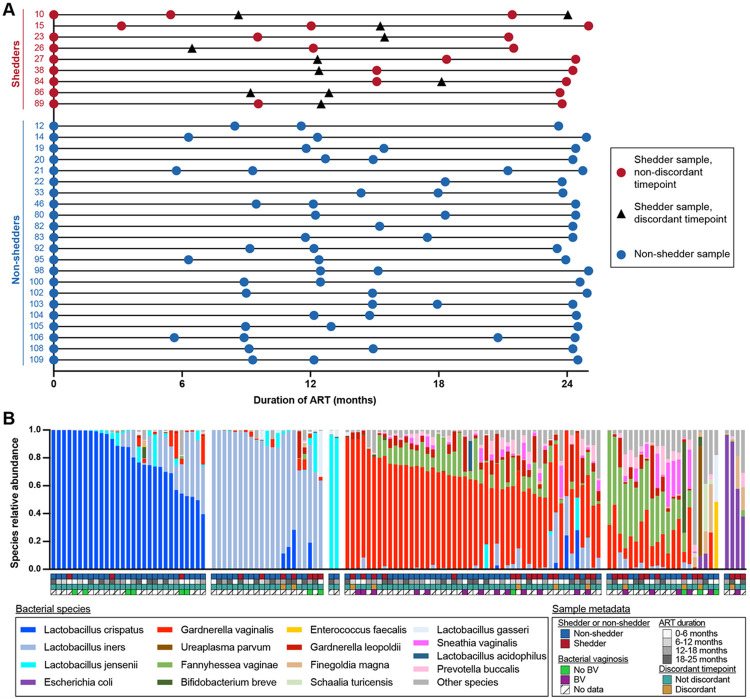
Cohort characteristics. A. Timeline of samples analyzed from each individual. Non-shedder samples are shown as blue circles, shedder samples from timepoints without discordant shedding are shown as red circles, and shedder samples from discordant shedding timepoints are shown as black triangles. B. Samples grouped by microbiome community cluster. Samples are shown as vertical bars colored by species relative abundance. Metadata is shown as rows of colored squares beneath the samples. By multinomial logistic regression, community clusters were not associated with ART duration or shedder/non-shedder status, except for cluster 3 which was associated with non-shedder status (p-values in Supplementary Table 2). Cluster 3 consists of two samples from the same non-shedder individual.

To investigate the roles of both the microbiome and virome in discordant HIV shedding, we analyzed 125 cervicovaginal specimens collected longitudinally from 31 WLHIV in Lima, Peru (Table 1, Fig. 1A, Supplementary Table 1). Specimens were collected quarterly from participants over a period of two years as part of a larger study characterizing discordant shedding in people living with HIV^4^. In this study, we analyzed an average of 4 specimens per participant. Most specimens (n = 120) were cervicovaginal lavages (CVL). When CVL samples were not available, vaginal swabs (n = 2), cytobrushes (n = 2), or TearFlo paper strips (n = 1) were used to sample the cervicovaginal environment. Nine women experienced discordant HIV shedding during the study period (shedders) and 22 did not (non-shedders). Non-shedders were older than shedders at study enrollment (non-shedder median age = 34 years, shedder median age = 28 years; Mann-Whitney test, p = 0.047) (Extended Data Fig. 1A). Shedders and non-shedders had similar CD4 counts at enrollment and similar CD4 recoveries when values from enrollment and after 2 years of ART were compared (Mann-Whitney test; p = 0.94 at enrollment, p = 0.94 at final visit, p = 0.94 for change from enrollment to final visit) (Extended Data Fig. 1B). Using metagenomic next-generation sequencing (NGS) after physically enriching for bacteria, we obtained a median of 15.6x10^6^ (IQR 13.7x10^6^ – 17.8x10^6^) raw read pairs and 1.95x10^6^ (IQR 8.7x10^5^ – 4.7x10^6^) quality-filtered, deduplicated individual reads per sample. Most samples clustered into groups dominated by *Lactobacillus crispatus* (n = 29), *Lactobacillus iners* (n = 21), *Gardnerella vaginalis* (n = 48), or a mixed community (n = 21), with a few samples dominated by *Lactobacillus jensenii* (n = 2) or *Escherichia coli* (n = 4) (Fig. 1B). There were no significant differences in bacterial alpha diversity, beta diversity, or cluster assignment between different sample types (CVL, vaginal swabs, cytobrushes or TearFlo strips) (Extended Data Fig. 1C-1F, Supplementary Table 2).

**Figure 2:**
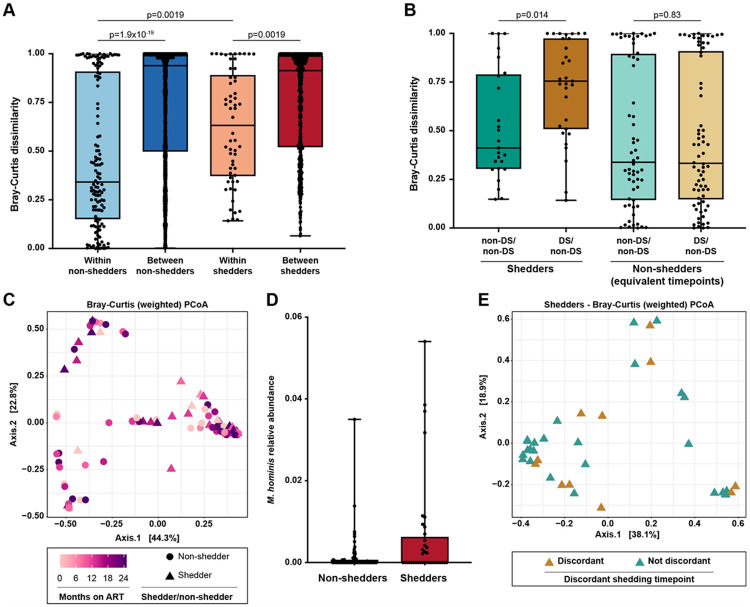
Bacterial microbiome in shedders and non-shedders. A. Bacterial species Bray-Curtis dissimilarity between pairs of samples from the same individual (within-individual comparisons) or from different individuals (between-individual comparisons). Statistical significance was assessed using the Mann-Whitney test and p-values were adjusted for multiple comparisons using the Benjamini-Hochberg method. B. Bacterial species Bray-Curtis dissimilarity between pairs of non-discordant samples within the same shedder individual (non-DS/non-DS), or sample pairs where one sample was from a discordant shedding timepoint and the other was from a non-discordant timepoint within the same individual (DS/non-DS). Equivalent timepoints from non-shedders serve as a control. Statistical significance was assessed using the Mann-Whitney test and p-values were adjusted for multiple comparisons using the Benjamini-Hochberg method. C. Principal coordinates analysis (PCoA) of shedder and non-shedder samples, using Bray Curtis dissimilarity calculated from bacterial species relative abundance. Samples are colored by duration of ART. Non-shedder samples are shown as circles and shedder samples are shown as triangles. Statistical significance was determined by PERMANOVA, p=0.33 for ART duration and p=0.34 for shedder/non-shedder status. D. *Mycoplasma hominis* relative abundance in non-shedders and shedders. Box bounds show the interquartile range and whiskers show the minimum and maximum. Statistical significance was determined by MaAsLin2, q=0.048. E. Principal coordinates analysis (PCoA) of shedder samples, using Bray-Curtis dissimilarity calculated from bacterial species relative abundance. Discordant shedding timepoints are colored brown, and non-discordant samples are colored green. Statistical significance was determined by PERMANOVA, p=0.85.

Intrapersonal microbiome variation (beta diversity) was lower than interpersonal variation, suggesting intraindividual stability over time (Fig. 2A; Bray-Curtis dissimilarity; Mann-Whitney test, p = 1.9x10^−19^ for non-shedders, p = 0.0019 for shedders). Shedders had higher intrapersonal microbiome beta diversity than non-shedders (Fig. 2A; Bray-Curtis dissimilarity, Mann-Whitney test, p = 0.0019). We tested the hypothesis that perturbation was most pronounced during discordant shedding by comparing diversity between discordant and non-discordant timepoints within individuals (Fig. 2B). Beta diversity was significantly higher between samples when one timepoint was discordant and the other was non-discordant, compared to pairs of non-discordant timepoints (Bray-Curtis dissimilarity; Mann-Whitney test, p = 0.014), suggesting that higher intrapersonal variation in shedders reflects dysbiosis during discordant shedding rather than prolonged fluctuations. Principal coordinates analysis (PCoA) using Bray-Curtis dissimilarity showed no association between microbiome composition and ART duration (PERMANOVA, p = 0.33) or shedder/non-shedder status (PERMANOVA, p = 0.34) (Fig. 2C), although discriminant analysis with MaAsLin2^50^ showed higher *Mycoplasma hominis* relative abundance in shedders compared to non-shedders (Fig. 2D, q = 0.048). PCoA of shedder samples showed no difference between discordant and non-discordant timepoints (Fig. 2E; PERMANOVA, p = 0.85). Microbiome community clusters were not associated with shedder/non-shedder status, ART duration, or discordant shedding timepoints (Fig. 1B, Supplementary Table 2). These results suggest that no specific microbiome composition is associated with shedder/non-shedder status, ART duration, or discordant shedding events, implying that higher variation during discordant shedding reflects individual-specific, not shared, dysbioses. To determine whether this could be explained by common functional characteristics encoded by bacterial genomes that might not be reflected in taxonomic profiles, we performed functional profiling with HUMAnN3^51^ (Extended Data Fig. 2A). We observed similar results for bacterial metabolic pathway and gene family beta diversity as for taxonomic beta diversity (Extended Data Fig. 2B-2I). 172 metabolic pathways were associated with microbiome community clusters but not with shedder/non-shedder status, discordant shedding events, or ART duration (MaAsLin2, Supplementary Table 3). Results were similar for gene families (Supplementary Table 4). This suggests that functional profiles mirror taxonomic profiles, with similar beta diversity dynamics during discordant HIV shedding.

### Non-shedders’ viromes are less stable than shedders’ viromes, with anellovirus and papillomavirus relative abundance changing over time.

**Figure 3:**
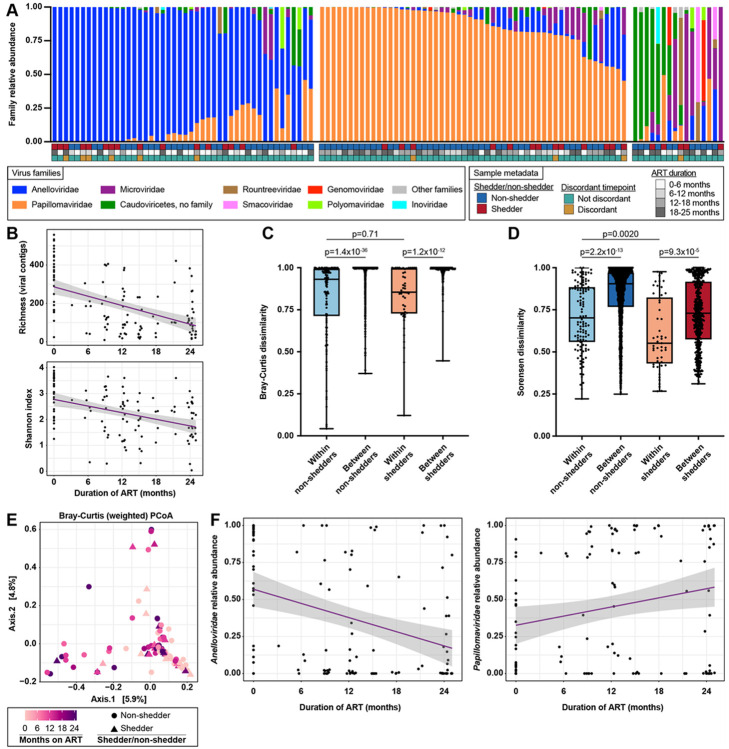
Virome over time in shedders and non-shedders. A. Samples grouped by virome community cluster. Samples are shown as vertical bars colored by family relative abundance. Metadata is shown as rows of colored squares beneath the samples. B. Virome alpha diversity (viral contig richness and Shannon index) by duration of ART. Shedder and non-shedder samples are plotted together. The linear regression line is shown in purple and the 95% confidence band is shown in gray. Statistical significance was assessed by linear mixed modeling (LME), p=7.7x10^−10^ for richness and p= p=8.3x10^−8^ for Shannon index. C. Viral Bray-Curtis dissimilarity (weighted) between pairs of samples from the same individual (within-individual comparisons) or from different individuals (between-individual comparisons). Statistical significance was assessed using the Mann-Whitney test and p-values were adjusted for multiple comparisons using the Benjamini-Hochberg method. D. Viral Sorensen dissimilarity (unweighted) between pairs of samples from the same individual (within-individual comparisons) or from different individuals (between-individual comparisons). Statistical significance was assessed using the Mann-Whitney test and p-values were adjusted for multiple comparisons using the Benjamini-Hochberg method. E. Principal coordinates analysis (PCoA) of shedder and non-shedder samples, using Bray Curtis dissimilarity calculated from virus contig relative abundance. Samples are colored by duration of ART. Non-shedder samples are shown as circles and shedder samples are shown as triangles. Statistical significance was determined by PERMANOVA, p=0.001 for ART duration and p=0.016 for shedder/non-shedder status. F. *Anelloviridae* relative abundance (left) and *Papillomaviridae* relative abundance (right) by ART duration. Shedder and non-shedder samples are plotted together. Linear regression lines are shown in purple and 95% confidence bands are shown in gray. Statistical significance was determined using MaAsLin2, q=8.8x10^−5^ for *Anelloviridae* and q=0.037 for *Papillomaviridae.*

Given the temporal dynamics of the bacterial microbiome, we examined the virome for similar patterns. After enrichment for virus-like particles (VLPs), we visualized VLPs in specimens using epifluorescence microscopy to corroborate VLP enrichment (Extended Data Fig. 3A). VLP concentrations ranged from 2.5x10^5^ – 2.2x10^8^ particles per mL of VLP-enriched supernatant, with a median of 1.1x10^7^ particles per mL (Extended Data Fig. 3B). Using metagenomic NGS of nucleic acids extracted from VLP-enriched supernatants and amplified by multiple displacement amplification (MDA), we obtained a median of 1.6x10^7^ (IQR 4.5x10^6^ – 2.2x10^7^) raw read pairs and 2.4x10^6^ (IQR 1.1x10^6^ – 3.5x10^6^) quality-filtered, deduplicated individual reads per sample. 12,574 unique contigs > = 500 nt were assembled from the samples and sequencing controls, and 1,596 were identified as viral. After mapping quality-filtered reads to the viral contigs, 116 samples had > 1,000 viral reads and were retained for further analysis. Samples clustered into groups dominated by *Anelloviridae* (n = 46), *Papillomaviridae* (n = 54), or bacteriophages including *Microviridae* and *Caudoviricetes* (n = 16) (Fig. 3A). Virome alpha diversity was inversely associated with ART duration (linear mixed modeling (LME), p = 7.7x10^−10^ for viral contig richness, p = 8.3x10^−8^ for Shannon index), with no significant difference between shedders and non-shedders (LME, p = 0.19 for richness, p = 0.90 for Shannon index) (Fig. 3B). Because MDA only amplifies DNA, we performed sequence-independent amplification (SIA) on the viral nucleic acids to amplify RNA and DNA. We found plant-infecting RNA viruses in a few samples, but no RNA viruses known to infect humans or bacteria, and most samples had no RNA viruses identified (Extended Data Fig. 4A). Therefore, we focused our analyses on the DNA virome using the MDA data.

Like bacterial microbiome variation, intrapersonal virome variation (beta diversity) was significantly lower than interpersonal variation (Fig. 3C, Bray-Curtis dissimilarity (weighted); Mann-Whitney, p = 1.4x10^−36^ for non-shedders, p = 1.2x10^−12^ for shedders; Fig. 3D, Sorensen dissimilarity (unweighted); Mann-Whitney, p = 2.2x10^−13^ for non-shedders, p = 9.3x10^−5^ for shedders). However, weighted intrapersonal variation was not significantly different between shedders and non-shedders (Fig. 3C, Bray-Curtis dissimilarity; Mann-Whitney, p = 0.71). Further, unweighted intrapersonal variation was significantly higher in non-shedders than in shedders (Sorensen dissimilarity; Mann-Whitney test, p = 0.0020) (Fig. 3D). In weighted (Bray-Curtis) PCoA, virome composition was associated with ART duration (PERMANOVA, p = 0.001) and shedder/non-shedder status (PERMANOVA, p = 0.016) (Fig. 3E). Because ART duration trended toward interaction with shedder/non-shedder status (PERMANOVA, p = 0.069), we carried out PCoA on shedders and non-shedders separately (Extended Data Fig. 5A). Virome composition was associated with ART duration in non-shedders (PERMANOVA, p = 0.001), and trended toward association with ART duration in shedders but was not significant (PERMANOVA, p = 0.072). In unweighted (Sorensen) PCoA of combined shedders and non-shedders, virus presence-absence was associated with ART duration (PERMANOVA, p = 0.001) but not shedder/non-shedder status (PERMANOVA, p = 0.63), with no interaction between ART duration and shedder/non-shedder status (PERMANOVA, p = 0.15) (Extended Data Fig. 5B). These results suggest that virome composition changes over time in WLHIV taking ART, with more pronounced changes among non-shedders.

**Figure 4:**
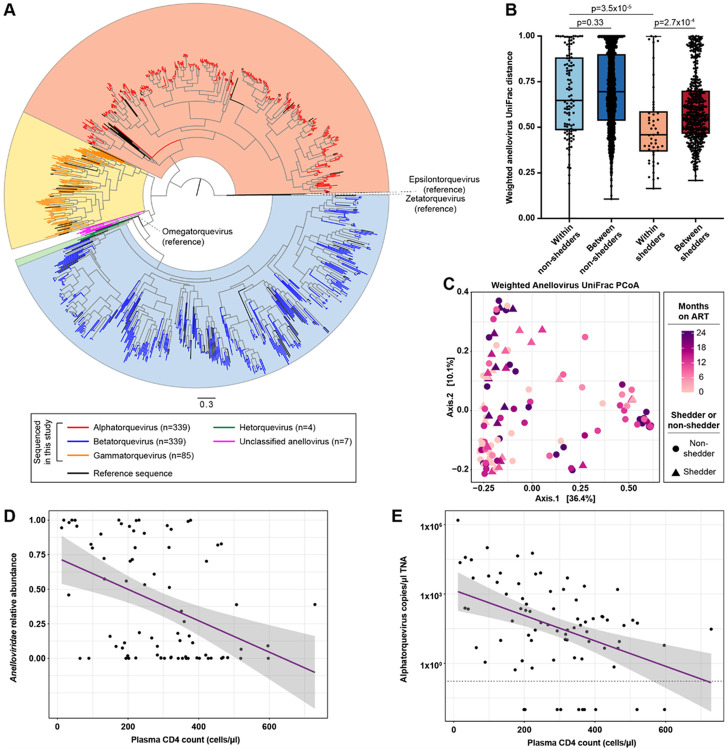
Anellovirus diversity over time in shedders and non-shedders. A. Anellovirus ORF1 amino acid phylogeny. Colored tips (red, blue, orange, green, and pink) represent sequences generated in this study. Reference sequences are shown in black. B. Weighted anellovirus UniFrac distance between pairs of samples from the same individual (within-individual comparisons) or from different individuals (between-individual comparisons). Statistical significance was assessed using the Mann-Whitney test and p-values were adjusted for multiple comparisons using the Benjamini-Hochberg method. C. Principal coordinates analysis (PCoA) of shedder and non-shedder samples, using weighted anellovirus UniFrac distance. Samples are colored by duration of ART. Non-shedder samples are shown as circles and shedder samples are shown as triangles. Statistical significance was assessed by PERMANOVA, p=0.005 for ART duration and p=0.81 for shedder/non-shedder status. D. Cervicovaginal *Anelloviridae* NGS relative abundance (top) and alphatorquevirus qPCR copy numbers (bottom) versus plasma CD4 counts. Copy numbers are expressed in copies/μl of extracted total nucleic acid (TNA). Shedder and non-shedder samples are plotted together. Linear regression lines are shown in purple and 95% confidence bands are shown in gray. The dotted horizontal line separates samples with detectable and undetectable alphatorquevirus by qPCR. Statistical significance was assessed by linear mixed modeling (LME), p=2.1x10^−4^ for *Anelloviridae* relative abundance and p=0.04 for alphatorquevirus copy numbers. To facilitate visualization on a log scale, a pseudocount of 0.01 copies/μl was added to all alphatorquevirus qPCR copy numbers, but LME analyses were based on the raw copy numbers.

We used differential abundance analysis (MaAsLin2^50^) to identify viruses responsible for changes in virome beta diversity over time. *Anelloviridae* relative abundance was inversely associated with ART duration (q = 8.8x10^−5^), while *Papillomaviridae* relative abundance increased over time (q = 0.037) (Fig. 4F). ART duration trended toward interaction with shedder/non-shedder status for several viral families, but these trends were not significant (Supplementary Table 5). When non-shedders were analyzed separately, *Anelloviridae* relative abundance decreased over time after ART initiation and *Papillomaviridae* relative abundance increased (Extended Data Fig. 5C). No taxa changed significantly over time in shedders. These results suggest that changes in virome composition after ART initiation are driven by *Anelloviridae* and *Papillomaviridae,* especially among non-shedders.

### Anellovirus communities change over time, with decreased alphatorquevirus abundance and increased betatorquevirus proportions.

Because the *Anelloviridae* family contributed significantly to the temporal virome signature (Fig. 3), we performed phylogenetic analysis of the anellovirus contig ORF1 gene sequences to better define anellovirus community dynamics. We obtained predicted ORF1 sequences from 751/799 anellovirus contigs. 21 contigs had more than one predicted ORF1 sequence, for a total of 774 sequences. At the genus level, most anelloviruses were classified as alphatorqueviruses (n = 339), betatorqueviruses (n = 339), or gammatorqueviruses (n = 85), with a few hetorqueviruses (n = 4) and sequences falling outside known anellovirus genera (n = 7) (Fig. 4A). Compared to all viral genera, relative abundance of alphatorqueviruses and incomplete anellovirus genomes (i.e., without ORF1) decreased over time after ART initiation (MaAsLin2, q = 0.0019 and q = 0.0019) (Extended Data Fig. 6A). There were no significant changes in the relative abundance of other anellovirus genera. This suggests that decreased *Anelloviridae* relative abundance reflects decreased *Alphatorquevirus* relative abundance.

We used the anellovirus phylogeny to calculate UniFrac distance, a beta diversity metric that accounts for phylogenetic distance. In shedders, intrapersonal anellovirus beta diversity was significantly lower than interpersonal diversity (weighted UniFrac, Mann-Whitney, p = 2.7x10^−4^), while for non-shedders there was no significant difference between intrapersonal and interpersonal anellovirus beta diversity (weighted UniFrac, Mann-Whitney, p = 0.33) (Fig. 4B). Intrapersonal variation was also significantly lower in shedders than non-shedders (Fig. 4B; Mann-Whitney, p = 3.5x10^−5^). Results were similar for unweighted UniFrac distance (Extended Data Fig. 6B). These results suggest that anellovirus communities are less stable over time in non-shedders compared to shedders. In PCoA using weighted anellovirus UniFrac distance, anellovirus community compositions changed significantly by ART duration (PERMANOVA, p = 0.005), with no significant difference between shedders and non-shedders (PERMANOVA, p = 0.81) (Fig. 4C). Because ART duration trended toward interaction with shedder/non-shedder status (PERMANOVA, p = 0.078), we carried out separate PCoA analyses on non-shedder and shedder samples. In these separate analyses, ART duration was associated with anellovirus community structure in non-shedders (PERMANOVA, p = 0.006), but not in shedders (PERMANOVA, p = 0.12) (Extended Data Fig. 6C). In unweighted anellovirus UniFrac PCoA of combined shedders and non-shedders, anellovirus presence-absence was associated with ART duration (PERMANOVA, p = 0.001) but not with shedder/non-shedder status (PERMANOVA, p = 0.59), with no interaction between ART duration and shedder/non-shedder status (PERMANOVA, p = 0.31) (Extended Data Fig. 6D). We performed differential abundance analyses (MaAsLin2) to identify specific anelloviruses responsible for changing anellovirus diversity over time. When shedders and non-shedders were analyzed together, *Betatorquevirus* relative abundance increased and relative abundance of partial anellovirus genomes decreased relative to other anellovirus genera (Extended Data Fig. 6E; MaAsLin2, q = 0.036 and q = 0.036). When non-shedders were considered separately, *Betatorquevirus* relative abundance increased (MaAsLin2, q = 0.028) (Extended Fig. 6F) and *Alphatorquevirus* relative abundance tended to decrease but did not reach significance (MaAsLin2, q = 0.060). No anellovirus genera changed significantly over time in shedders. Together with the whole-virome analyses, where *Alphatorquevirus* relative abundance decreased over time in the virome as a whole, these results suggest that betatorqueviruses consequently make up a larger proportion of the remaining anellovirus community, and these changes are more pronounced among non-shedders.

We corroborated the NGS data with quantitative PCR (qPCR) specific to the untranslated region of the alphatorquevirus genome (Extended Data Fig. 7A). Alphatorquevirus genome copy numbers were correlated with *Alphatorquevirus* NGS relative abundance (linear regression, p = 0.041) and trended toward association with *Anelloviridae* relative abundance (linear regression, p = 0.085), but not *Betatorquevirus* or *Gammatorquevirus* relative abundance (linear regression, p = 0.96 and p = 0.84, respectively) (Extended Data Fig. 7B). Alphatorquevirus copy numbers tended toward inverse association with ART duration, but this was not significant (LME, p = 0.090) (Extended Data Fig. 7C), and there was no significant difference between shedders and non-shedders (LME, p = 0.51).

Because anellovirus abundance has previously been associated with immune status^44,52-59^, we considered whether increasing CD4 counts could explain decreasing anellovirus relative abundance. CD4 counts were inversely associated with *Anelloviridae* relative abundance (LME, p = 1.1x10^−4^) (Fig. 4D), *Alphatorquevirus* relative abundance (Extended Data Fig. 7D, LME, p = 1.7x10^−4^) and alphatorquevirus qPCR copy numbers (Fig. 4E; LME, p = 0.04). There was no association between CD4 counts and *Betatorquevirus* or *Gammatorquevirus* relative abundance (LME, p = 0.77 and p = 0.36, respectively) (Extended Data Fig. 7D). When we analyzed non-shedders separately, *Anelloviridae* NGS relative abundance, *Alphatorquevirus* NGS relative abundance and alphatorquevirus qPCR copy numbers were inversely associated with CD4 counts (LME; p = 2.1x10^−4^, p = 1.3x10^−4^, and p = 0.04, respectively), but there were no significant associations in shedders (Extended Data Fig. 7E). These results suggest a relationship between immune status and anellovirus abundance, specifically alphatorqueviruses, in the FGT.

### Papillomavirus populations are stable over time in shedders and non-shedders.

**Figure 5:**
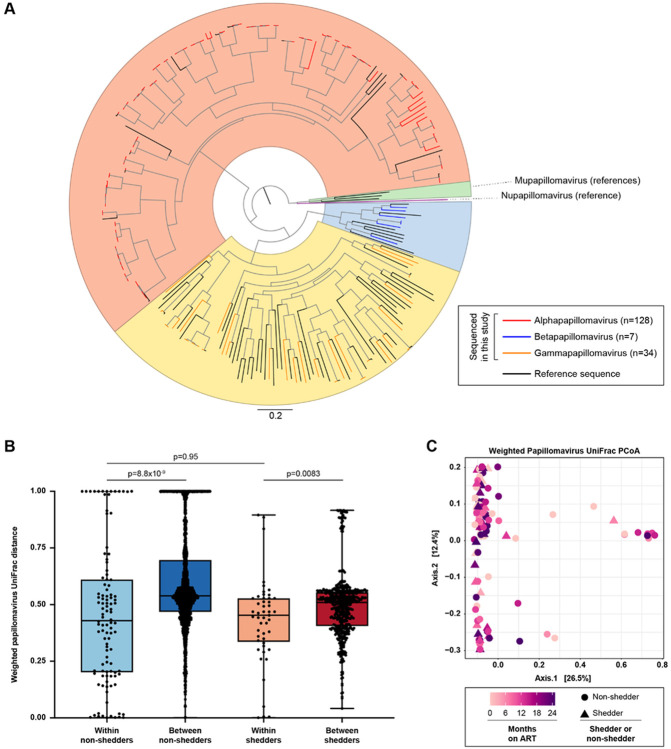
Papillomavirus diversity over time in shedders and non-shedders. A. Papillomavirus E1-E2-L2-L1 nucleotide phylogeny. Colored tips (red, blue, and orange) represent sequences generated in this study. Reference sequences are shown in black. B. Weighted papillomavirus UniFrac distance between pairs of samples from the same individual (within-individual comparisons) or from different individuals (between-individual comparisons). Statistical significance was assessed using the Mann-Whitney test and p-values were adjusted for multiple comparisons using the Benjamini-Hochberg method. C. Principal coordinates analysis (PCoA) of shedder and non-shedder samples, using weighted UniFrac distance calculated from papillomavirus phylogeny and relative abundance. Samples are colored by duration of ART. Non-shedder samples are shown as circles and shedder samples are shown as triangles. Statistical significance was determined by PERMANOVA, p=0.33 for ART duration and p=0.28 for shedder/non-shedder status.

Because the *Papillomaviridae* family also contributed to the temporal virome signature (Fig. 3), we conducted phylogenetic analysis of the concatenated papillomavirus contig E1, E2, L2, and L1 genes to characterize papillomavirus diversity over time. We obtained E1-E2-L2-L1 sequences from 169/190 papillomavirus contigs. Most were alphapapillomaviruses (n = 128), with some betapapillomaviruses (n = 7) and gammapapillomaviruses (n = 34) (Fig. 5A). *Alphapapillomavirus* relative abundance increased over time (Extended Data Fig. 8A, MaAsLin2, q = 0.044), while other papillomavirus genera did not change significantly.

Unlike anellovirus beta diversity, both non-shedders and shedders had lower intrapersonal papillomavirus beta diversity compared to interpersonal diversity, with no significant difference between shedders and non-shedders (Fig. 5B; weighted papillomavirus UniFrac distance, Mann-Whitney, p = 8.8x10^−9^, p = 0.0083, and p = 0.95, respectively). Unweighted papillomavirus beta diversity results were similar (Extended Data Fig. 8B). In PCoA using papillomavirus UniFrac distance, there were no significant differences by ART duration or shedder/non-shedder status (Fig. 5C, Extended Data Fig. 8C; PERMANOVA; weighted, p = 0.33 and p = 0.28, respectively; unweighted, p = 0.18 and p = 0.10, respectively). These results suggest that papillomavirus diversity is stable over time in shedders and non-shedders.

## DISCUSSION

We identified intraindividual and longitudinal patterns in the cervicovaginal microbiome and virome of WLHIV with and without discordant HIV shedding. Specifically, the bacterial microbiome was less stable over time in shedders. Virome composition changed significantly in association with ART duration, with more pronounced changes among non-shedders.

Higher intraindividual bacterial beta diversity in shedders suggests more frequent changes in microbiome composition compared to non-shedders. This was driven by shedding timepoints, suggesting that microbiome alterations may be associated with shedding events. By contrast, lower intraindividual diversity in non-shedders suggests stability over time. No specific microbiome composition was associated with shedders or shedding events, suggesting that stability over time is more important than specific taxa in the context of discordant shedding. This is interesting because previous studies have often focused on specific microbiome community states or bacterial taxa as markers of FGT health^11^. Previous analysis of this cohort linked discordant shedding with proliferation of infected cells, rather than HIV replication^4^. Other studies have identified associations between cervicovaginal microbiota and T cell populations in the FGT^14^. Thus, the microbiome may contribute to discordant shedding by influencing the number of infected cells in the FGT, that may proliferate in response to disruption of the FGT. Alternatively, the microbiome may be influenced by other factors (trauma through intercourse, inflammation, etc.) that also contribute to discordant shedding.

We identified significant longitudinal changes in the cervicovaginal virome. Specifically, *Anelloviridae* and *Alphatorquevirus* relative abundance decreased and overall anellovirus community diversity changed during two years of ART. Anellovirus relative abundance and alphatorquevirus absolute abundance measured by qPCR were inversely associated with plasma CD4 counts. Anelloviruses are prevalent in healthy adult humans and are not known to be pathogenic^49^. Interestingly, numerous studies have associated anellovirus loads with immune suppression, including in people living with HIV^44,52-58^ and in organ transplant recipients^49,59^. Thus, the anellovirus dynamics that we observed may reflect participants’ immune recovery during two years of ART. The fact that the associations were more pronounced among non-shedders may be a consequence of sampling size, or may suggest underlying immune dysregulation in shedders.

While *Papillomaviridae* relative abundance increased over time, papillomavirus diversity did not change significantly. Because relative abundance data is compositional, papillomavirus relative abundance may have been inflated when anellovirus relative abundance decreased, with no underlying change in papillomavirus viral load. Although Phi29 polymerase may preferentially amplify small circular single-stranded DNA^60^, our alphatorquevirus qPCR absolute copy numbers were consistent with NGS relative abundance, suggesting that our results represent an actual biological signature. Besides plant virus reads identified in a few samples, we did not find any RNA viruses by sequence-independent amplification (SIA). This could mean that no RNA viruses were present in the specimens, or the RNA could have degraded during storage.

Our cohort consisted of ART-naïve women from a clinical setting who were initiating government-sponsored ART in Lima, Peru. Because all the study participants received ART, we cannot directly compare our results with data from WLHIV not receiving ART. While this comparison would be biologically informative, such a study would be incompatible with current treatment standards. To our knowledge, only a few studies have analyzed the cervicovaginal microbiome or virome in women from Peru^61-63^. Therefore, our data contributes valuable information about the FGT microbiome and virome in women from this region. Prevalence of vaginal microbiome community states varies between cohorts, with some studies reporting differences between women from different geographic regions or different racial and ethnic backgrounds^11-15,19,64-66^. Therefore, studying diverse groups of WLHIV worldwide would indicate whether our results are generalizable to other populations.

This longitudinal analysis of the cervicovaginal virome and microbiome in WLHIV identifies intrapersonal and temporal patterns associated with discordant HIV shedding and ART duration. These findings support further investigation of the role of bacterial communities in discordant HIV shedding in women with suppressed plasma viral loads, as well as the role of anelloviruses during immune suppression and recovery.

## METHODS

### Specimens:

All study participants provided informed consent. This study was approved by the Institutional Review Boards of the Hospital Dos de Mayo (Lima, Peru), Seattle Children's Hospital (Seattle, Washington, USA), and Arizona State University (Tempe, Arizona, USA). Cervicovaginal specimens were collected as part of a 18-24-month study evaluating low-level viremias (aka “blips”) and discordant genital shedding in ART naïve men and women from Lima, Peru^4,67^. Specimens were collected every three months over a two-year period for each participant, and plasma CD4 counts were measured every six months. Cervicovaginal lavages were collected by washing the uterine cervix with sterile 1X phosphate buffered saline (PBS) and collecting the 1X PBS from the vaginal fornix as described^68^. Plasma and CVL HIV RNA were quantified by qRT-PCR as described^69^. Discordant shedding was assessed in participants with ART suppression, defined as median plasma HIV RNA below the limit-of-quantification (1.48 log_10_ copies(**c**)/mL), without virologic failure (HIV RNA > 3.0 log_10_c/mL), who had HIV RNA detected in the CVL (1.48 log_10_ copies(**c**)/mL) for the women. Nine women experienced discordant HIV shedding (shedders) and 22 women did not experience discordant shedding (non-shedders). To select timepoints for microbiome and virome analysis, non-shedders were matched with shedders based on average CD4 counts over the duration of ART (2–4 non-shedders for each shedder). We analyzed the microbiome and virome at enrollment (month 3 for one shedder who did not have an enrollment specimen available); for shedders, discordant shedding timepoints and immediate pre- or post- shedding timepoints, as available; matched visits for non-shedders; and at 24 months (month 21 for two shedders who did not have month 24 specimens available) (Fig. 1A). When CVL specimens were not available, we analyzed other available specimen types: vaginal swabs (n = 2), cytobrushes (n = 2), or TearFlo paper strips (n = 1).

### Sample processing

Specimens were thawed on ice and mixed by pipetting. Up to 800 μl of CVL were centrifuged for 10 minutes at 7,000xg at 4° C. The supernatants were used immediately for virus-like particle enrichment and virome sequencing, and the pellets were frozen at 80° C until used for bacterial microbiome sequencing. PBS negative controls (800 μl) were processed alongside the samples through microbiome and virome sequencing to assess contamination. In the case of vaginal swabs, cytobrushes, or TearFlo strips, 1 mL of 1X PBS was added to the specimen and vortexed for 30 seconds, and 800 μl was then processed in the same way as the CVLs.

### Bacterial microbiome sequencing

Frozen bacterial pellets (CVL samples and 1X PBS controls) were thawed on ice. DNA was extracted with the DNeasy PowerSoil Pro kit (Qiagen; Germantown, MD), following the manufacturer’s instructions modified to include 775 μl of Solution CD1, 20 minutes bead beating in a TissueLyser II, 3 minutes of centrifugation at 16,000 g to dry the spin column membrane, and elution in 60 μl of Solution C6. DNA concentrations were measured on a NanoDrop spectrophotometer, and 5–25 μl of DNA (approximately 100–500 ng DNA) per sample were used as input for the Illumina DNA Prep library kit, following the manufacturer’s protocol with 6 cycles for the indexing PCR. Two μl of ZymoBIOMICS Microbial Community DNA Standard was used to build a positive control library. Libraries were pooled, quantified with a Qubit fluorometer and sequenced (paired-end, 2x150 bp) on a NextSeq 1000/2000 sequencer (Illumina; San Diego, CA). Depending on the number of reads obtained per library, some libraries were re-sequenced and the results concatenated to obtain a minimum of 10 million read pairs per sample.

### Virus-like particle enrichment and virome sequencing:

CVL supernatants and PBS controls were passed through a 0.2-μm pore filter to exclude bacterial and eukaryotic cells. Filtrates were treated with benzonase and Baseline-ZERO DNase to digest non-encapsidated nucleic acids. Total nucleic acids (TNA) were then extracted from 500 μl of the treated filtrates on the bioMérieux eMAG, with PBS added as needed if filtrates were below the required volume. Viral DNA was amplified by multiple displacement amplification (MDA) using Phi29 DNA polymerase (GenomiPhi V2 (Cytiva; Marlborough, MA)), with the kit’s lambda phage DNA for a positive control. The amplified DNA was variably diluted (up to 1:10 depending on band brightness after gel electrophoresis) and used as input for the Illumina DNA Prep library kit, with 6 cycles for the indexing PCR. Libraries were individually quantified with a Qubit fluorometer, pooled and sequenced on an Illumina NextSeq 2000 sequencer (paired-end, 2x150 bp). Separately, we performed sequence-independent amplification (SIA) of viral RNA and DNA by first-strand cDNA synthesis with SuperScript IV reverse transcriptase (ThermoFisher; Waltham, MA) and primers 5'-GTTTCCCAGTCACGATCNNNNNNNNN-3' and 5'-GTTTCCCAGTCACGATC-3', second strand DNA synthesis with DNA Polymerase I Klenow fragment (New England Biolabs; Ipswich, MA), and amplification with AccuPrime Taq high fidelity polymerase (ThermoFisher; Waltham, MA) and primer 5'-GTTTCCCAGTCACGATC-3'. We used cell-culture derived rhinovirus B14 RNA as a positive control. The SIA products were used undiluted as input for the DNA Prep library kit (Illumina; San Diego, CA) and sequenced in the same way as the MDA products.

### Virus-like particle enumeration by fluorescence microscopy:

Unextracted, nuclease-treated filtrates remaining from the virus-like particle (VLP) enrichment were frozen at 80° C until processed for VLP enumeration by fluorescence microscopy, using an adaption of previously described methods^70,71^. Fifteen-100 μl of CVL filtrate and PBS negative controls were mixed with 10 mL sterile SM buffer and filtered onto a 0.02-μm pore, 25 mm diameter Anodisc aluminum oxide filter (Cytiva; Marlborough, MA). De-identified, remnant stool specimens (ASU IRB record STUDY00011967) processed similarly to the CVL samples (resuspension in SM buffer, 0.45-μm and 0.2-μm filtration, and nuclease treatment) and myxoma virus with a known titer were used as positive controls. Filters were dried, stained with 25X SYBR Gold (ThermoFisher; Waltham, MA), and mounted on slides in 15 μl of glycerol/PBS buffer with 0.1% ascorbic acid (working stock: 1 mL glycerol, 980 μl PBS and 20 μl 10% ascorbic acid). Slides were visualized at 60X with oil immersion on an AX R confocal microscope (Nikon; Tokyo, Japan) with a 488-nm wavelength laser. Five fields were randomly selected per sample, and NIS-Elements software (Nikon; Tokyo, Japan) was used to count particles between 50 nm and 500 nm in size. VLP counts for each sample were averaged across the number of fields viewed and used to calculate VLP concentrations in the CVL filtrates and controls.

### Bacterial microbiome analysis

Bacterial sequencing reads were quality-filtered, sequencing adapters, PhiX and human sequences removed, reads deduplicated and paired reads merged using BBTools (v. 38.38)^72^. Taxonomy was assigned to the clean reads with KrakenUniq (v. 0.7.3)^73^ using a custom database (RefSeq archaeal, bacterial, viral, plasmid, and fungal genomes, plus the GRCh38 human genome; downloaded in January 2022). To reduce false positives, we masked taxa that were assigned fewer than 1,800 unique kmers per million reads. For downstream analyses, we parsed the read counts at the species level, i.e., we included reads that could be classified at the species level or lower and excluded reads that could only be classified at the genus level or higher. We excluded probable false positive taxa (*Cyanobacteria/Melainabacteria* and *Rickettsiales*), as well as eukaryotes, archaea, viruses, and plasmids. Contaminant bacterial taxa were identified using decontam (R package v. 1.18.0)^74^ and removed at a threshold of 0.1 (*Acidovorax* sp. KKS102, *Streptococcus equi,* and *Cutibacterium acnes*). To normalize for different sequencing depths between samples, species read counts were expressed as number of reads per 100,000 clean reads in a sample, or as relative abundance where appropriate.

Bacterial alpha diversity was measured by species richness, calculated as the number of taxa present in each sample, and Shannon index, calculated using vegan (R package, v. 2.6-4)^75^. Bacterial beta diversity was measured by Bray-Curtis dissimilarity (weighted) and Sorensen dissimilarity (unweighted), calculated with vegan using species relative abundance and species presence-absence, respectively. PCoA analyses were conducted using phyloseq (R package, v. 1.42.0)^76^. Microbiome community groups were defined by kmeans clustering using the R stats package (v. 4.2.2)^77^ on species relative abundance with k = 6. MaAsLin2^50^ was used on species relative abundance to identify species differentially associated either with ART duration or shedder/non-shedder status.

For functional profiling, we assigned reads to bacterial gene families and metabolic pathways using HUMAnN3^78^. Contaminant identification and beta diversity analyses were performed as described above, using normalized gene family and metabolic pathway read counts or relative abundance for input as appropriate. We used MaAsLin2 to identify gene families and pathways associated with sample metadata and microbiome community clusters.

### Virome analysis

MDA and SIA virome data were analyzed separately. For the MDA data, sequencing adapters were trimmed with Cutadapt (v. 4.0)^79^. Reads were quality-filtered, PhiX and human reads removed, reads deduplicated, and paired reads merged with BBTools. Contigs were assembled with metaSPAdes (v. 3.15.4)^80^ and deduplicated with cd-hit-est (v. 4.8.1)^81^ at 95% identity across 95% of contig length. We retained contigs ≥500 bases in length for further analysis.

### Identification of viral contigs

We used Cenote-Taker2 (v. 2.1.5)^82^ with the standard database and VirSorter2 (v. 2.2.4)^83^ to identify candidate viral contigs. We queried the candidate viral contigs against the NCBI NT database (downloaded June 2020) with megablast (BLAST + v. 2.13.0)^84^ and removed contigs with ≥95% nucleotide identity and query coverage to human sequences. We used CheckV (v. 1.0.1)^85^ to check for false positive, non-viral sequences. We considered contigs to be viral if they were classified as complete, high quality or medium quality by CheckV; or if they were low-quality with at least three viral genes and no more than one host gene; or if they were classified as proviruses. For contigs classified as proviruses, we kept only the viral regions and discarded flanking host sequences. We discarded contigs flagged by CheckV as having high kmer frequency, or which had been classified as bacteria by Cenote-Taker2 during candidate contig identification. To assign taxonomy, we queried the viral contigs against a custom viral protein database (NCBI RefSeq and neighbor viral sequences, downloaded January 2023) using blastx (e-value 1x10^−3^) and used the taxonomy of the best hit in the database. We discarded contigs classified as *Mimiviridae* as likely false positives. We used Bowtie 2 (v. 2.5.1)^86^ to map the quality-filtered sample reads against the viral contigs. Contaminant contigs were identified using decontam (threshold = 0.1) and removed (six contigs). For downstream analyses, we kept samples that had at least 1,000 viral reads (116 of 125 samples (93%)).

### Ecological analyses

Virome alpha (Shannon index and richness) and beta diversity metrics (Bray-Curtis and Sorensen dissimilarity) were calculated and PCoA was conducted as described for the bacterial microbiome, using viral contig relative abundance for weighted analyses and presence-absence for unweighted analyses.

Kmeans clustering was conducted on virus family relative abundance in R as described for the bacterial microbiome, with k = 3. MaAsLin2 was used at different taxonomic levels (family, genus, and contig) to identify viral taxa associated with ART duration or shedder/non-shedder status.

#### Anellovirus phylogenetic analysis:

We used Geneious Prime (v. 2023.0.4, http://www.geneious.com) to predict open reading frames (ORFs) from the anellovirus contigs called by blastx during taxonomy assignment. ORFs ≥1,000 nucleotides in length were extracted, manually curated, translated to amino acids and aligned with reference *Alphatorquevirus, Betatorquevirus, Gammatorquevirus, Hetorquevirus, Omegatorquevirus, Epsilontorquevirus* and *Zetatorquevirus* sequences retrieved from GenBank (accessions in Supplementary Table 6) using MAFFT (v. 7.505)^87^ with auto algorithm selection. The alignment was trimmed with trimAl (v. 1.4.rev15)^88^ using the -gappyout option and a maximum likelihood phylogeny was constructed with IQ-TREE (v. 2.0.3)^89^ with the model LG + F + G4 and 1,000 bootstrap replicates. The phylogeny was visualized and rooted in FigTree (v. 1.4.4)^90^ using *Epsilontorquevirus/Zetatorquevirus* as the outgroup, and contigs were assigned to genera based on clade location. Phylogeny-based genus assignments, rather than blastx-based genus assignments, were used for anellovirus contigs in genus-level virome analyses (e.g., MaAsLin2 analyses). For anellovirus UniFrac distance, a separate phylogeny was built with the contig ORF1 sequences and a single *Zetatorquevirus* reference sequence, using the method described above. This phylogeny was re-rooted in R using ape (v. 5.6-2)^91^, the reference sequence was pruned using phyloseq (v. 1.42.0)^76^, and weighted and unweighted UniFrac distance were calculated with phyloseq using the pruned phylogeny and anellovirus contig relative abundance. PCoA analyses were performed using weighted and unweighted UniFrac distance based on the pruned phylogeny and anellovirus contig relative abundance or presence-absence.

### Papillomavirus phylogenetic analysis

We used Geneious Prime to predict ORFs from the papillomavirus contigs called by blastx during taxonomy assignment. We mapped contig ORFs ≥1,000 nucleotides in length against reference *Alphapapillomavirus, Betapapillomavirus, Gammapapillomavirus, Mupapillomavirus* and *Nupapillomavirus* genomes and annotated the ORFs based on mapping to the reference genes. After manual curation, concatenated contig E1-E2-L2-L1 nucleotide sequences and reference *Alphapapillomavirus, Betapapillomavirus, Gammapapillomavirus, Mupapillomavirus* and *Nupapillomavirus* sequences retrieved from RefSeq (accessions in Supplementary Table 7) were aligned with MAFFT with auto algorithm selection, trimmed with trimAl with the -gappyout option, and a maximum likelihood phylogeny constructed with IQ-TREE with the model GTR + G and 1,000 bootstrap replicates. The phylogeny was visualized in FigTree and rooted using *Mupapillomavirus/Nupapillomavirus* as the outgroup. Contigs were assigned to genera based on their clade, and phylogeny-based genus assignments were used for papillomavirus contigs in genus-level virome analyses. Papillomavirus beta diversity analyses were performed in the same way as for anelloviruses, with a separate phylogeny built from the contig E1-E2-L2-L1 sequences and a single *Mupapillomavirus* reference.

### SIA virome analysis

SIA virome sequencing reads were processed through the same quality control pipeline as described for the MDA virome reads. SIA reads were then queried against the viral RefSeq and neighbor sequences database using blastx (e-value 1x10^−3^) and parsed with MEGAN (community edition, v. 6.24.21)^92^.

### Alphatorquevirus qPCR assay

Forward primer 5’-GTGCCGIAGGTGAGTTTA-3’, reverse primer 5’-AGCCCGGCCAGTCC-3’ and probe 5’-[6-FAM]-TCAAGGGGCAATTCGGGCT-[TAMRA]-3’ were used with Applied Biosystems 2X TaqMan Fast Universal PCR Master Mix and 5 μl of sample total nucleic acid (TNA) per 25-μl reaction. Reactions were performed on an Applied Biosystems QuantStudio 7 Flex Real-Time PCR system (ThermoFisher; Waltham, MA), with reaction conditions of 95° C for 20 seconds and 40 cycles of 95° C for 1 second and 60° C for 20 seconds.

### Statistical analysis

All statistical analyses were conducted in R (v. 4.2.2)^77^. P values < 0.05 were considered statistically significant. Where appropriate, corrections were made for multiple comparisons using the Benjamini-Hochberg method^93^. We corrected for repeated sampling using participant ID as a random effect, where appropriate. PERMANOVA was performed using the adonis2 function in the vegan package (v. 2.6-4)^75^. Multinomial logistic regression was done with the mblogit function in the mclogit package (v. 0.9.6)^94^. Differential abundance analysis was conducted using MaAsLin2 (v. 1.12.0)^50^. Linear mixed modeling was done using the nlme package (v. 3.1–160)^95^. Details of statistical models can be found in the GitHub code repository for this study. Plots were generated using R, GraphPad Prism (v. 9.3.1, www.graphpad.com) and FigTree.

## Figures and Tables

**Table 1:** Cohort characteristics

	Shedders	Non- shedders	Statistical significance
Number of participants	9	22	N/A
Number of specimens analyzed per participant: median (interquartile range)	4 (4–4)	4 (4–4)	p = 0.52
Age at enrollment: median (interquartile range)	28 (24–30)	34 (30–39)	p = 0.047
CD4 at enrollment: median (interquartile range)	132 (89–223)	141 (51.25–205.5)	p = 0.94
CD4 after 2 years of ART: median (interquartile range)	454 (307.5–510)	356.5 (295.5–415.5)	p = 0.94
CD4 change from initial to final visit: median (interquartile range)	209.5 (140.25–304.5)	208 (152–329.75)	p = 0.94

Statistical significance was assessed by the Mann-Whitney test, with the Benjamini-Hochberg method used to correct for multiple comparisons where appropriate. Number of specimens, age, and CD4 counts are expressed as median and interquartile range.

## Data Availability

De-identified sequencing reads with human sequences removed have been deposited to the NCBI Sequence Read Archive under PRJNA1077994.

## Code availability

Code used in this study can be found at https://github.com/ASU-Lim-Lab/CVL-Microbiome-HIV-Discordant-Shedding.
